# Gold-Modified Molecularly Imprinted *N*-Methacryloyl-(*l*)-phenylalanine-containing Electrodes for Electrochemical Detection of Dopamine

**DOI:** 10.3390/bioengineering9030087

**Published:** 2022-02-22

**Authors:** Nemah Abu Shama, Süleyman Aşır, Mehmet Ozsoz, Ilgım Göktürk, Deniz Türkmen, Fatma Yılmaz, Adil Denizli

**Affiliations:** 1 Department of Analytical Chemistry, Faculty of Pharmacy, Near East University, Nicosia 99138, Cyprus; nemahmahmoudmohammad.abushama@neu.edu.tr; 2 Department of Materials Science and Nanotechnology Engineering, Near East University, Nicosia 99138, Cyprus; 3 Department of Biomedical Engineering, Faculty of Engineering, Near East University, Nicosia 99138, Cyprus; mehmet.ozsoz@neu.edu.tr; 4 Department of Chemistry, Faculty of Science, Hacettepe University, Beytepe, Ankara 06800, Turkey; ilgim@hacettepe.edu.tr (I.G.); denizt@hacettepe.edu.tr (D.T.); denizli@hacettepe.edu.tr (A.D.); 5 Chemistry Technology Division, Vocational School of Gerede, Bolu Abant Izzet Baysal University, Bolu 14900, Turkey; fyilmaz71@gmail.com

**Keywords:** dopamine, gold nanoparticles, pencil graphite electrode, molecularly imprinted polymer, differential pulse voltammetry, cyclic voltammetry

## Abstract

A molecularly imprinted polymer-based pencil graphite electrode (MIP PGE) sensor, modified with gold nanoparticles, was utilized for the detection of dopamine in the presence of other biochemical compounds using cyclic voltammetry (CV) and differential pulse voltammetry (DPV), depending on its strong electroactivity function. The pulse voltammetry methods recorded the highest response. In addition to the high oxidation rate of DA and the other biomolecule interferences available in the sample matrix used, which cause overlapping voltammograms, we aimed to differentiate them in a highly sensitive limit of detection range. The calibration curves for DA were obtained using the CV and DPV over the concentration range of 0.395–3.96 nM in 0.1 M phosphate buffer solution (PBS) at pH 7.4 with a correlation coefficient of 0.996 and a detection limit of 0.193 nM. The electrochemical technique was employed to detect DA molecules quantitatively in human blood plasma selected as real samples without applying any pre-treatment processes. MIP electrodes proved their ability to detect DA with high selectivity, even with epinephrine and norepinephrine competitor molecules and interferences, such as ascorbic acid (AA). The high level of recognition achieved by molecularly imprinted polymers (MIPs) is essential for many biological and pharmaceutical studies.

## 1. Introduction

Dopamine (DA) denotes one of the essential monoamine neurotransmitters (chemical messengers between neurons) found naturally in beings, which is currently being studied extensively. It is usually spread in the brain tissue of the central nervous system of the human body, which has various important roles, especially inside the brain, including executive functions, blood flow, reinforcement, motivation, behavior, and reward [1]. As a result, irregular DA levels may precipitate various disorders with complex functions, such as Alzheimer’s disease, depression, Parkinson’s disease, and others [2]. Numerous analytical methods, such as spectroscopy, fluorescence analysis [3], capillary electrophoresis [4], and high-pressure liquid chromatography [HPLC] [5], have been used for the detection of DA. Nevertheless, these procedures are high-cost, complex, tedious, and require special equipment, even though the spectrophotometry technique has recently been improved to be detectable and straightforward with a high limit of detection [6].

The electrochemical methods overcome others’ drawbacks, such as the high sensitivity due to the low limit of detection, low time consumption, and cheap equipment needed. In the past few years, because of DA’s electroactivity feature, the detection of it by electrochemical procedures has been developed [7,8,9]. To obtain a highly selective detection of DA among other interferences and to obtain remarkable outcomes with high peak resolution and sensitivity, sensitive methods, such as CV and DPV, were chosen to carry out the DA measurement and detection; however, in the biological matrixes, the concentration of DA is very low. Hence, the CV and DPV techniques provide a potential to enhance the electrochemical electrode sensor performance with low back current interferences. DA is a highly oxidative compound, which makes dealing with it challenging during the voltammetry procedure, so caution should be taken. Many studies detected certain biochemical compounds, such as uric acid (UA) and ascorbic acid (AA), which are available in a human fluid along with DA in very high concentrations [10,11]. Because they are oxidized at different peak potentials of 558 mV for UA and 152 mV for AA, simultaneous analysis of these compounds is also possible [12]. Hence, many electrodes were modified to improve their selectivity and sensitivity of response to DA, such as modified electrodes with nanocomposites [13], nanoparticles [14], and pyrolytic graphite electrodes [15]. Even though these modified electrodes have differentiated the overlay peaks to a specific level and reached immediate DA detection with other biochemical compounds, the limit of detection of DA in a real sample is still high. Moreover, due to the high reactive oxidation properties of DA, it can easily react with other compounds, such as ascorbic acid, that exist in the real sample, which affects the accuracy of recognition [16]. Thus, a highly selective and sensitive technique is required to determine DA in the presence of other biological compounds.

Molecularly imprinted polymer (MIP) electrodes indicate one of the main sensors used to detect many biochemical compounds in a highly sensitive and selective approach, which has various applications in many criteria in voltametric detection research [17]. MIP electrodes demonstrated that they might be investigated for their ability to detect DA with high accuracy despite existing biological compound interferences [18]. By using a surface plasmon resonance sensor based on MIP’s, sensitive and selective dopamine measurements with a correlation coefficient of 0.98 in the biological samples were successfully performed [19]. The double detections approach was improved over DA aptamer with MIPs upon reduced graphene oxide and gold NPs, with a detection limit of 4.7 × 10^−8^ M associated with a single detection material (aptamer or MIP) [20]. Molecularly imprinted solid-phase extraction was applied to separate DA from the human urine by using HPLC as a validation technique [21]. For the bio-analysis, the functional monomer 3-aminophenyl boronic acid (APBA) was used for the electro-polymerization step in MIP synthesis for the DA recognition and applied in electro-chemiluminescence (ECL) [22]. Dopamine was also detected as an interference in the existence of AA using a boron-doped diamond (BDD) film electrode to avoid overlapping in the differential pulse voltammetry method, but with a high detection limit [23]. A gold electrode with a Fullerene-C_60_ modification was also utilized to identify the presence of DA in the occurrence of excess AA by using the square wave voltammetry (SQWV) method in a physiological pH media [24]. Nevertheless, a high limit of detection was achieved.

This study tried to achieve a highly sensitive and selective determination of DA in the MIP electrode cell system. In this system, the important step is the recognition of DA at a very low detection limit. Though there are many reports conducted on the modified MIP sensors, this present work describes the application of the modified electrode by the use of an *N*-methacryloyl-(*l*)-phenylalanine (MAPA)-containing polymer to produce the pencil graphite electrode surface and accelerate the electron transfer mechanism to perform with a higher sensitivity for DA molecules. Detecting other DA with its other neurotransmitter competitors, such as epinephrine (EP) and norepinephrine (NE), was achieved in a design lower than 0.05 g/L. In addition, the recognition of DA molecules in a highly complex medium such as human blood plasma was also achieved as an application of original samples.

## 2. Materials and Methods

### 2.1. Materials and Reagents

Dopamine hydrochloride (DA, 98%), epinephrine hydrochloride (EP, 98%), norepinephrine hydrochloride (NE, 98%), (L)-phenylalanine methyl ester, (L)-cysteine methyl ester, ethylene glycol dimethacrylate (EDMA), 2-hydroxyethyl methacrylate (HEMA), methanol, and acetic acid were supplied from Merck (Darmstadt, Germany). The chemicals used to prepare buffers in this research were sodium dihydrogen phosphate adjusted with phosphoric acid to obtain a pH of 1.5, acetic acid and sodium acetate for the preparation of acetate buffer to obtain a pH of 5.5, and potassium phosphate monobasic and potassium phosphate dibasic for the preparation of phosphate buffer to achieve a pH of 7.4. Ammonium chloride was adjusted by ammonia to obtain a pH of 8.5, and, finally, carbonic acid and sodium hydroxide adjusted the pH to 10.5, which were obtained from Merck (Darmstadt, Germany). Deionized (DI) water (18.2 MΩ cm) was utilized to make all of the aqueous solutions and treated with pure lab Ultra Analytic (ELGA Lab Water, Runcorn, UK). The pH meter was used to measure the pH, and the ultrasonic water bath was also used in solution preparation. Pencil graphite electrodes (PGEs) were used in pencil tips (Tombow 0.5, HB) obtained from local stationery stores.

### 2.2. Apparatus

The AUTOLAB-PGSTAT204 potentiostat/galvanostat with NOVA 2.1.2 software (Metrohm, Utrecht, The Netherlands) performed DPV and CV measurements. The electrochemical pulse measurements for both techniques were carried out using an electrochemical cell consisting of three main electrodes. The first electrode, called a counter electrode (CE), or auxiliary electrode, was a platinum wire. The second electrode was the reference electrode (RE), made of Ag/AgCl (3M KCl). The third one was the working electrode (WE). The solutions were freshly prepared before each measurement for each DA concentration to avoid any oxidation reaction of the analyte of concern.

Furthermore, all detection methods were verified at a room temperature of 25 °C. The parameters used for both pulse voltametric techniques, DPV and CV, were adjusted for measurements. The upper and lower vertex potentials for the CV method were +0.6 V and 0.4 V, respectively, whereas the number of scans and the scan rate were 1 and 0.05 V/s, respectively. The DPV was carried out under a start potential and stop potential of 0 V and 0.4 V, respectively, in addition to the step height of 0.005 mV and the amplitude of 0.025 V in a time interval of 0.5 s. All received data and peaks were evaluated with OriginPro8 (Northampton, MA, USA), and Microsoft Excel 2013 (Redmond, WA, USA). Centrifuge and sonicator devices were used for solution and sample preparation. A Century India Ltd. digital pH meter (Model CP-901, Chandigarh, India) was employed for pH optimization.

### 2.3. Preparation of Stock Solutions and Real Sample

The buffer solution was first prepared using potassium phosphate and potassium dihydrogen phosphate to adjust the pH to the physiological pH of 7.4. Then, the DA standard stock solution was prepared by dissolving the proper amount of DA.HCl powder in an adequate amount of D.H_2_O and phosphate buffer solution (PBS) to the adjusted volume used for the dilution part later on. The same procedure was carried out to prepare the EP and NE solutions by dissolving an appropriate amount of each in PBS. After obtaining permission from the Institute Ethical Clearance Committee, human blood samples were collected from volunteers at NEU hospital. The blood samples were centrifuged at about 1000 rpm for 5 min. Using the DPV approach at the modified MIP PGE sensor, the supernatant blood plasma was used to determine the DA levels. The blood plasma samples were spiked with different concentrations of DA at pH 7.4 before analysis.

### 2.4. Production of Gold Nanoparticles

The Turkevich method was used to synthesize the gold nanoparticles (AuNPs) using gold (III)chloride trihydrate (HAuCl_4_·3H_2_O) salt, which is reduced to Au with sodium citrate [25,26]. In brief, into the 100 mL of boiling water, preheated sodium citrate solution (12 mL, 1%, *w*/*w*) and 8.5 mg of HAuCl_4_ salt were added. This solution was heated by vigorously stirring until the color turned deep red. In the final step, the prepared solution of AuNPs was cooled, and the volume of the final solution was adjusted to 100 mL. The average gold core diameter (D) and standard deviations were calculated for each nanoparticle sample by averaging the particle sizes obtained from the TEM images. The TEM image of the AuNP sample was obtained using a Tecnai G2 F30 S-TWIN is a 300 kV/FEG transmission electron microscope (FEI Tecnai-G2-F30, Hillsboro, OR, USA). The accelerating voltage used in the measurement was 300 keV. After determining the average diameter of the AuNPs using the TEM image, the concentration of AuNPs was computed [27]. Firstly, the number of Au atoms per nanoparticle (N) was calculated using Equation (1), while Equation (2) was used for calculating the AuNP concentration, C. In the equations, ρ stands for Au density (fcc, 19.3 g/cm^3^), M stands for Au atomic weight, and D stands for the diameter of AuNPs, while N_T_ stands for the total number of Au atoms, V for the solution volume, and N_A_ for Avogadro’s number.

(1) $$N=\frac{\pi }{6}\frac{{\rho D}^{3}}{M}$$

(2) $$C=\frac{N_{T}}{{\mathrm{NVN}}_{A}}$$

A dynamic light scattering (DLS) analysis was conducted to estimate the hydrodynamic size of the sample of AuNPs by the Nano Zetasizer instrument (NanoS, Malvern Instruments, London, UK).

### 2.5. Synthesis of MAPA and MAC Functional Monomers

The functional monomer, *N*-methacryloyl-(*l*)-phenylalanine (MAPA), was selected to interact with the DA molecules. The practical method explained elsewhere in [28] was followed. In brief, 5.0 g of L-phenylalanine methyl ester and 0.2 g of NaNO_2_ were liquefied in 30 mL of K_2_CO_3_ aqueous solution (5%, *w*/*v*). Afterwards, this solution was chilled to 0 °C. Under nitrogen gas, 4.0 mL of methacryloyl chloride was gently added to this solution, then magnetically stirred for about 2 h at room temperature. The solution’s pH was adjusted to 7.0 at the last step of the chemical reaction. Subsequently, the solution was isolated using ethyl acetate as an organic solvent. In a rotating evaporator, the aqueous phase was then evaporated. Finally, cyclohexane and ether solvents were used for the crystallization of MAPA.

According to a previously published method, the synthesis of *N*-methacryloyl-(*l*)-cysteine (MAC), the functional monomer, was carried out [29]. In a few words, 0.2 g NaNO_2_ and 5.0 g of (L)-cysteine methyl ester were dissolved in 30 mL of a prepared 5% *v*/*v* K_2_CO_3_ aqueous solution and refrigerated to 0 °C. Next, 4.0 mL of methacryloyl chloride was gently added into the NaNO_2_ aqueous solution under nitrogen. After that, the solution was magnetically stirred for about 2 h at 22 °C. After the reaction mixture was neutralized at pH 7.0, the unreacted methacryloyl chloride was isolated using ethyl acetate. In a rotating evaporator device, the aqueous phase was evaporated. In ethanol:ethyl acetate with a ratio of 1:1 (*v*/*v*), the residue (MAC) was crystallized and collected.

### 2.6. Preparation of Molecularly Imprinted Polymer-Based Pencil Graphite Electrode (MIP PGE) Sensor

MIP PGE Sensor was prepared using PGE 0.5/HB pencil tips. The functional MAC monomer was mixed with AuNPs (0.05 mmol:0.05 nmol) to prepare the MAC-AuNPs pre-complex. The complex formation of MAC monomers with AuNPs was measured using a UV-VIS spectrophotometer. In addition, MAPA and DA (0.2 mmol/0.02 mmol) were reacted for 1 h to formulate the *N*-methacryloyl-(*l*)-phenylalanine-Dopamine (MAPADA) pre-complex, and an EDMA (2 mmol) and HEMA (0.4 mmol) monomer mixture was added to the pre-complexed monomers. Lastly, two prepared mixtures and 5 mg of AIBN were added together and mixed for 1 h. To fabricate the MIP PGE sensor, tips were immersed into the prepared monomer mixture. UV light was used to start the polymerization process and allowed 1 h on the tips for converting the monomer mixtures to polymeric films. After the polymerization was completed, the DA was removed from the matrices using a methanol/acetic acid (80/20, *v/v*) mixture as a desorption agent. A UV-VIS spectrophotometer was used to measure the desorbed DA in the desorption solution until no DA absorption was detected at 285 nm [30]. A non-imprinted polymer-based pencil graphite electrode (NIP PGE) sensor was produced using a similar process, except for the addition of DA, a template molecule. NIP PGE sensor was used for the control experiments.

### 2.7. Characterization of MIP PGE and NIP PGE Sensors

The MIP PGE and NIP PGE sensors were characterized (wavenumber range of 400–4000 cm^−1^) using a Fourier transform infrared spectrophotometer with an attenuated total reflection (FTIR-ATR) spectrophotometer (Thermo Fisher Scientific, Nicolet iS10, Waltham, MA, USA). With the Kruss DSA100 contact angle (CA) instrument (Hamburg, Germany), the surface characterization of the MIP PGE and NIP PGE sensors was examined. Using the sessile drop method, the CA measurements of the MIP PGE and NIP PGE sensors were calculated from different parts of the sensor surfaces. DSA2 software was used to calculate the average drop angles. The surface morphology of the MIP PGE and NIP PGE sensors was also estimated by a scanning electron microscope (JSM-6400, JEOL, Akishima, Tokyo, Japan). The samples were coated with a thin gold-palladium (Au-Pd) alloy coating.

## 3. Results and Discussion

### 3.1. Characterization Studies

**Figure 1:**
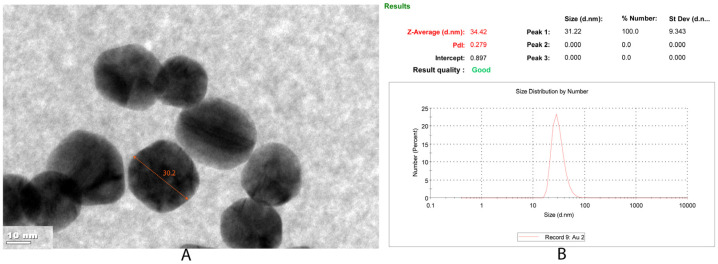
(**A**)TEM image and (**B**) DLS analysis of AuNPs.

The MAC monomer was mixed with AuNPs to form the MAC-Au pre-complex. The developed MAC-Au pre-complex was successfully integrated into the polymeric composition of the MIP PGE and NIP PGE sensors. The concentration of AuNPs was approximately measured by a TEM image of the AuNPs. The estimated diameter from the TEM measurements was 31.64 ± 4.43 nm. Figure 1A shows the TEM image of the AuNPs. After determining the average diameter of the AuNPs by using the TEM image, the concentration of the AuNPs was computed [27]. Using the average AuNP diameter calculated from the TEM images, the concentration of the AuNP solution was estimated to be 10.1 × 10^−7^ M.

The average size of the AuNPs (Z-average) measured by DLS analysis indicated the hydrodynamic diameter of the AuNPs. Figure 1B shows that monodisperse particles with a uniform average nanoparticle size distribution were produced (PDI = 0.279). Following measurements repeated 3 times (*n* = 3), the average size of the nanoparticles was determined to be 34.42 nm. As shown in Figure 1, the precise diameter measurement obtained by the TEM image was correlated with the DLS measurement results.

In the same way, the ratio of the functional monomer MAPA to the template DA molecule was 1:1 (0.1 mmol:0.1 mmol). After the complexation of the functional monomers MAC and MAPA with Au and DA, the formed pre-complexes were reacted with an EDMA (2 mmol) crosslinker and a HEMA (0.4 mmol) monomer to form MIP PGE and NIP PGE sensors. The polymerization technique was applied to prepare the NIP PGE sensors except for the DA molecules.

When the FTIR-ATR spectrum of the non-imprinted polymer (NIP) and molecularly imprinted polymer (MIP) was compared, similar signals were recorded because functional –C_6_H_5_, –NH_2_, and –OH groups are present in both the MIP and NIP polymeric structures. After the complexation of DA, the shifting of some functional groups was recorded. For example –C=O shifting from 1729 cm^−1^ to 1726 cm^−1^ and amide I band shifting from 1452 cm^−1^ to 1449 cm^−1^ in the NIP indicates that the DA molecule was integrated into the MIP structure (Supplementary Figure S1).

**Figure 2:**
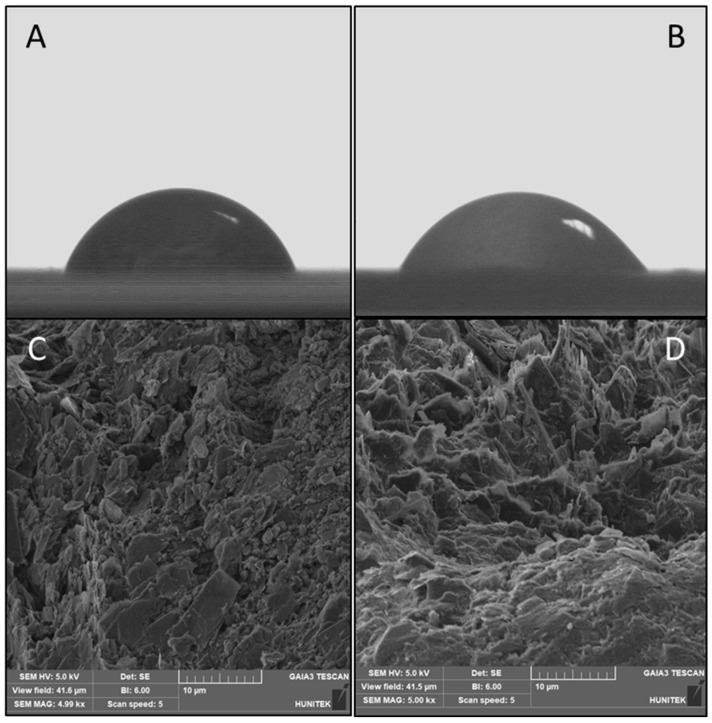
The surface characterization images of the MIP PGE and NIP PGE sensors. (**A**) CA image of MIP PGE sensor. (**B**) CA image of NIP PGE sensor. (**C**) SEM micrograph of MIP PGE sensor. (**D**) SEM micrograph of NIP PGE sensor.

Figure 2 represents the surface characterization images of the MIP PGE and NIP PGE sensors. The CA images of the MIP PGE and NIP PGE sensors are shown in Figure 2A,B. The approximated CA value for the MIP PGE sensor was 69.3° ± 2.52, while for the NIP PGE sensor it was 57.5° ± 1.98. As the hydrophobicity of the MIP PGE sensor surface enhanced as a result of the DA coordination to MAPA by molecular imprinting, the CA increased.

An SEM imaged the surface morphology of the MIP PGE and NIP PGE sensors at 5kx magnification values for each PGE. Figure 2C, D show the SEM images of MIP PGE and NIP PGE sensors, respectively.

### 3.2. Optimization and Kinetic Analysis by MIP PGE Sensor

**Figure 3:**
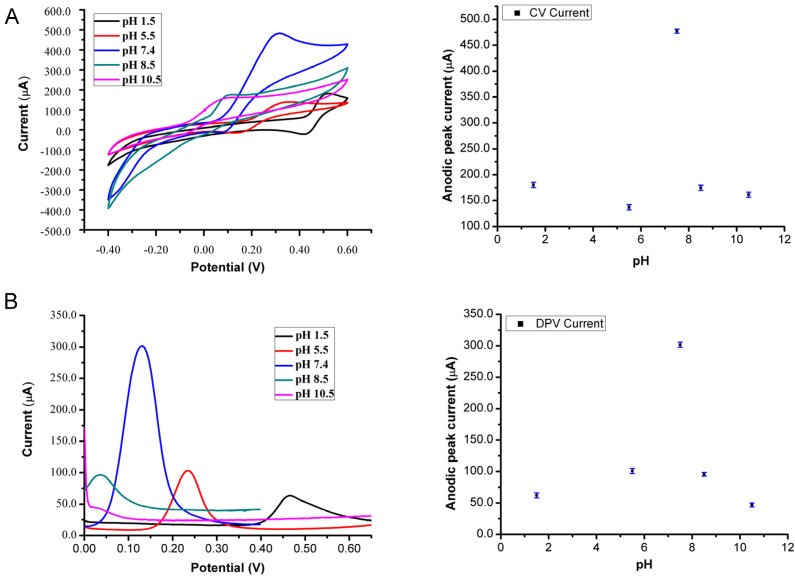
Optimization of the working pH for DA by voltammetry methods in different buffers at room temperature. (**A**) The detection of DA by CV in the different pH ranges of buffer solutions; (**B**) The detection of DA by DPV in the different pH ranges of buffer solutions.

**Figure 4:**
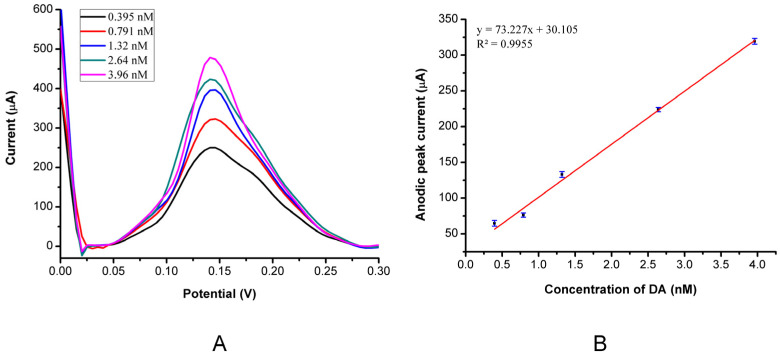
Optimization of DA and kinetic parameters. (**A**) Voltammograms of the MIP PGE sensor response for DA concentrations by DPV; (**B**) Calibration of DA concentration in a range of (0.395–3.96 nM).

Since DA in blood in the human brain has a pH of 7.2 to 7.4, optimization is needed to find out the highest electroactivity of DA in the electrochemical techniques. Hence, buffers were prepared with different pH values (1.5, 5.5, 7.4, 8.5, and 10.5) using phosphate, acetate, and ammonium solutions. The standard stock of DA at a concentration of 189.6 mg/mL was prepared with double distilled water and PBS at the same pH and stored in the refrigerator at 4 °C. Figure 3 indicates the detection of DA at different pH values, followed by the response of each voltammetric technique used with PGE 0.5/HB as a WE in the electrochemical cell. In both CV and DPV, the maximum voltammogram peak height was at the pH of 7.4, more than the others, which indicates the protonated form of DA compound, which alters the electroactivity of its moieties to the potential applied. Besides, the position of the peak potential was found to be shifted to lower positive potentials with the variation of pH (Supplementary Figure S2). In Figure 4, different concentrations of DA were prepared from the stock solution for the calibration curve, ranging from (0.395–3.96 nM) with PBS buffer solution.

The kinetic analysis was organized via the MIP PGE sensor through five different concentrations of DA (0.395, 0.791, 1.32, 2.64, and 3.96 nM) were prepared in 0.1 M PBS in 7.4 pH media. The concentrations were detected by the same DPV technique with start and stop potentials of 0 and 0.4, respectively, in a modulation time of 0.05 s. In addition, between each measurement, the desorption solution, which contains methanol:acetic acid in the appropriate amount of (9:1) *v*/*v* solution, was used to desorb the DA molecule from the MIP PGE sensor cavities, hence increasing the electrode affinity. The measurements were repeated three times (*n* = 3) to calculate the relative standard deviation, RSD%, and repeatability accuracy.

### 3.3. DPV Response of the MIP PGE Sensor for Different DA concentrations

The modified polymer electrode surface, which is negatively charged, helped in adsorbing the DA molecules of positive charge, which enhanced the electrode selectivity to the chemical of interest. The amino acid part of the MAPA monomer is phenylalanine, which comprises a basic amino group (−NH_2_) and an acidic carboxyl group (−COOH). Therefore, the negative charge in the modified polymer electrode surface derivatized with MAPA originates from the amino (−NH_2_) and the carboxylic acid (−COOH) functional groups of the phenylalanine part of the MAPA monomer. Phenylalanine has a pI of 5.48 (pKa1: 2.58 and pKa2: 9.24). Deprotonation of the acid functionalities increases with increasing pH of the mobile phase. Phenylalanine is negatively charged at the 0.1 M PBS media (pH: 7.4) (pI: 5.48). Figure 4 shows the voltammograms of five different DA concentrations (0.395 nM–3.96 nM) obtained by DPV, a highly sensitive technique among other analytical techniques. The potential peak was observed around ~0.15 V, where the increase of DA concentration enhanced the oxidation of DA.

For the analytical behaviour study, a linear correlation was observed between the DPV response current and the DA concentration used, ranging from 0.395 nM–3.96 nM. The detection limit was achieved as 0.193 nM (Table 1), which is better than other reported studies [31]. These results show that the *N*-methacryloyl-(*l*)-phenylalanine monomer can enhance electrode selectivity for DA molecules. Furthermore, the stability and sensitivity of the MIP PGE sensor to DA were also tested by time. The peak height (current) remained 97%, even after 10 uses.

### 3.4. Estimation of Selectivity and Imprinting Efficiency

The selectivity evaluation for the MIP PGE sensor was conducted by detecting other neurotransmitters with similar chemical structure and behavior to DA, such as the NE and EP molecules. To display the MIP PGE sensor, 1.32 nM of DA, NE, and EP were prepared in 0.1 M PBS at a pH of 7.4. The same procedure was used to analyze the competing behaviour of both molecules, and the same technique used in the kinetic study was also applied. To observe the imprinting effect, the neurotransmitters, with a concentration of 1.32 nM, were applied to both the MIP PGE and NIP PGE sensors, and the selectivity was evaluated. MIPs, acting as a recognition element, are used to create imprinted sites that are similar to the template molecules in size and shape. These imprinted sites act selectively and increase efficiency in the rebinding of target molecules [32]. As seen in Table 2, the MIP PGE sensor was 2.76 and 5.15 times more selective for the target DA molecule than the EP and NE molecules, respectively.

The availability of DA concentration in the human plasma is at a very low point. The many interference compounds are available in the matrix, which indicates an adequate reason for a crucial need for a selectivity measurement. Consequently, a simple, cheap, effective, and selective DA recognition technique should be established. For this reason, the MAPA monomer, which contains phenylalanine, of this MIP PGE sensor can determine the DA among other neurotransmitters molecules, even in micro-molar concentration levels of DA.

**Figure 5:**
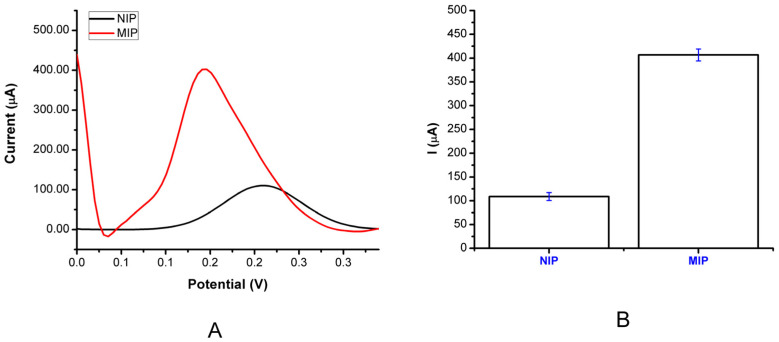
Modified electrode responses; (**A**) MIP PGE and NIP PGE sensor responses for the DA molecule; (**B**) Error bars for the MIP PGE and NIP PGE sensor responses (*n* = 3).

**Figure 6:**
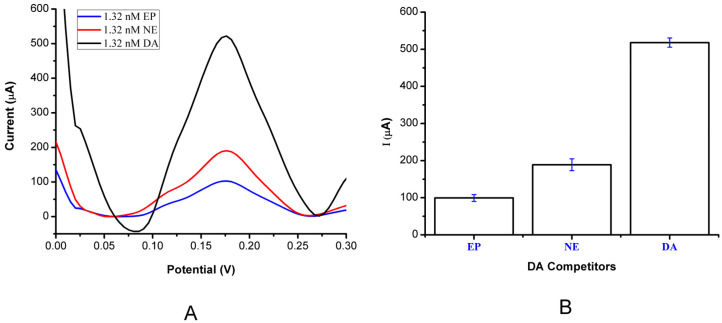
Selectivity study. (**A**) MIP PGE sensor response for DA (1.32 nM), NE (1.32 nM), and EP (1.32 nM) competitors; (**B**) Error bars for the MIP PGE sensor responses (*n* = 3).

The MIP electrode, as a recognition element, is used to produce the imprinted cavities identical to the template molecule in both shape and size. These imprinted sites are performing the selectivity function and enhancing the effectiveness of the analyte rebinding process. Figure 5 illustrates the selectivity evaluation of the DA detection by the MIP PGE sensor. The imprinting efficiency was determined by comparing the MIP PGE sensor with the NIP PGE sensor for detecting DA (1.32 nM DA), as shown in Figure 6. Since the catecholamines indicate the same main functional group, for the neurotransmitters such as DA, NE, and EP, there is a challenge to be differentiated by the MIPs electrode sensor, so it is clear that the three neurotransmitters are very similar in structure and differ only in one branched hydroxyl and methyl group attached to the R chain. In order to overcome this obstacle and to obtain a high selectivity by MIPs, chemical modification on the electrode surface was conducted to adjust the electrode surface kinetics toward detecting only the DA molecules rather than the others. During the synthesis process, some accomplished functional monomer materials that have a specific binding to the DA analyte compared to the other neurotransmitters were added to the working electrodes, to act somewhat like the lock–key combination, with only specific binding cavities to increase the electrochemical detection selectivity of DA in the existence of both NE and EP. Thus, The DA selectivity study of the MIP PGE sensor was performed using EP and NE, which were chosen as competitor molecules. On behalf of this aim, 1.32 nM NE and 1.32 nM EP solutions were examined with the MIP PGE sensor. The EP and NE chosen as competitor molecules were determined by comparing the MIP PGE sensor to the NIP PGE sensor for detecting DA (1.32 nM DA), as shown in Figure 6. The DA selectivity study of the MIP PGE sensor was performed using EP and NE chosen as competitor molecules. The results showed that the prepared sensor was 1.45 times more selective than EP and 1.90 times more selective than NE molecules. The calculated imprinting factor, reported as IF: 5.06, implied that the imprinting process was successfully performed. Moreover, DA was detected more selectively by the MIP PGE sensor than the NIP PGE sensor. The selectivity parameters were tabulated, as shown in Table 2.

### 3.5. Repeatability of MIP PGE Sensor

**Figure 7:**
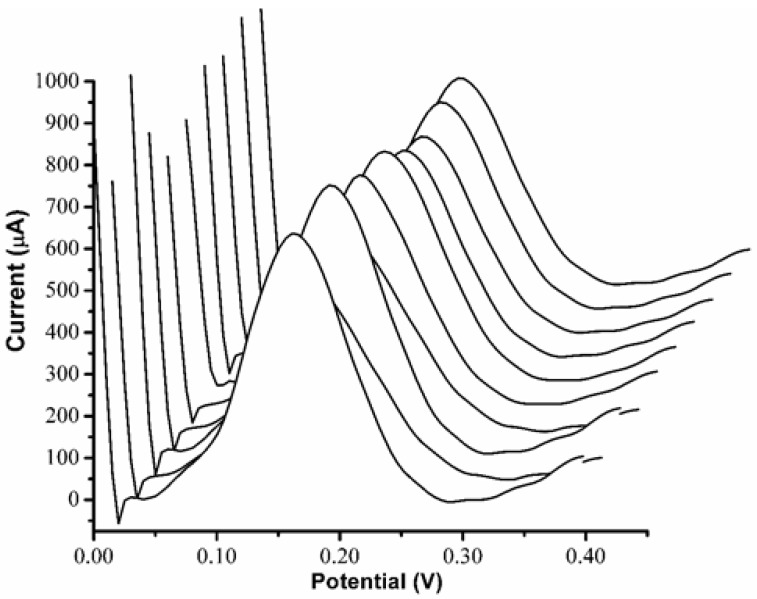
Repeatability study of the MIP PGE sensor. The response of the sensor electrode was for 5.27 nM DA. The measurements were repeated 10 times (*n* = 10).

The electrode surface modification with a polymer for the recognition of components is restricted due to the fouling of the electrode surface during the measurement and regeneration stage. At the same time, passivation of the three-dimensional cavities on the electrode surfaces created for the biological analyte of interest during these processes are also possible. The stability of the electrode structure used in this study can cause the pencil graphite electrode to be reusable by protecting the graphite surface against the cruel environment and working conditions. The repeatability of the MIP PGE sensor was tested by utilizing a 5.27 nM solution, and the MIP PGE sensor response was schemed as I (current) versus (V) potential applied, as shown in Figure 7.

As proof for the stability of the MIP PGE sensor used for DA detection, the MIP PGE sensor was immersed into the freshly prepared DA solution (5.27 nM) and detected by the DPV method 10 times (*n* = 10). The desorption step by immersing the electrode in the desorption solution of methanol and acetic acid in a ratio of (1:9, *v*/*v*) was carried out between each measurement. The voltammograms obtained for the 10 measurements were evaluated through a ratio equation as follows:

Peak. _Ratio_ = Peak._h (n−1)_/Peak._h (n)_ (3)

In Equation (3), h refers to the peak height of the measured signal after detection and n refers to the number of measurements carried out. The experimental outcomes showed that the obtained current (I) signal gave a relative standard deviation of 3.44% for DA, signifying a high stability in the electrodes. The modified electrodes were used 10 times to detect the same concentration of DA, and it was observed that the efficiency of the MIP PGE sensor was 97%. This shows that the MIP PGE sensor can be reusable up to 10 times without losing efficiency and affinity. Calculating the relative standard deviation RSD% value, which is around 4.131%, shows the high stability of the electrodes for the biological compound of interest.

### 3.6. DA Determination in a Real Biological Sample

**Figure 8:**
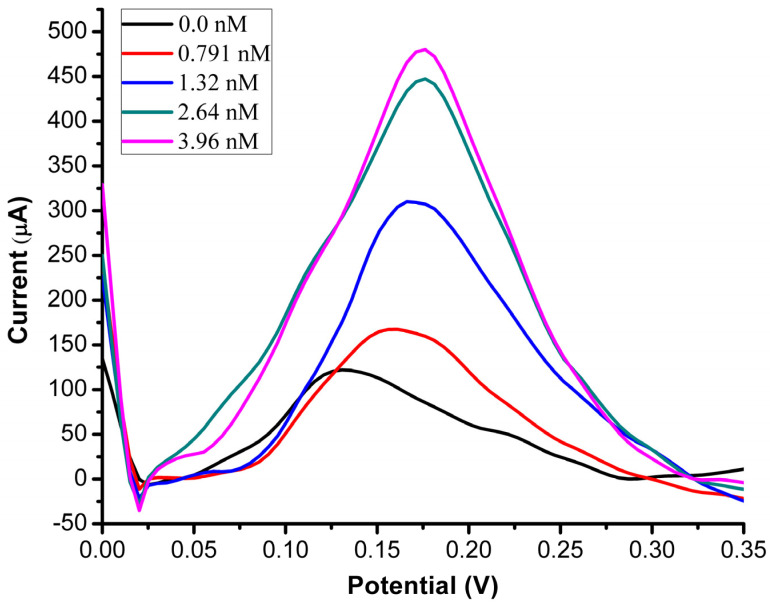
Voltammograms of spiked plasma samples ranging from 0.0 nM to 3.96 nM DA.

The synthesized MIP PGE sensor was prepared to recognize DA molecules from other molecules with similar functional groups (catecholamine) in human plasma samples. Furthermore, MIP can determine the DA molecules with high selectivity and sensitivity. The voltammetric method was applied to detect the DA molecules in human blood plasma. According to a recent study, the healthy human blood plasma DA level was 23.1 nM [33]. To measure the DA concentration level, the MIP PGE sensor was preserved with human blood plasma solutions spiked with different concentrations of DA solutions prepared at PBS pH 7.4, to reach the final DA concentrations of 0.395, 0.791, 1.32, 2.64, and 3.96 nM. As shown in Figure 8, a slight shift (~0.13 V) occurred in the non-spiked sample, due to the natural occurrence of NE and EP molecules in the serum plasma used. The change in peak height (current response) occurred due to the increase in DA concentrations used for spiking the plasma samples. The lowest peak height indicates the plasma sample without adding the DA solution. When comparing the currents obtained here and in Figure 4A, the current was different, due to the availability of matrix effect and interferences in the real biological sample analyzed in Figure 8, which reduced the current responses obtained in each sample. It is also clear from Figure 8 that as the concentration of DA increased the matrix effect diminished.

This study aims to develop and improve MIP PGE sensor to detect DA in prepared aqueous solutions and human plasma samples. The use of MAPA on the electrode surface enhanced the sensitivity for detecting DA, even in ultra-trace amounts in samples. MIPs supply very attractive functional chemical groups with imprinted sites for the particular selectivity of the molecule template. In comparison with other recognition materials, such as antibodies and enzymes, the method of MIP synthesis is much easier, has a lower cost, and has less time needed, which overcome many known disadvantages, in addition to the high recognition ability of DA molecules, even in a complicated matrix, which is essential for many biological studies [34,35].

The MAPA monomer, with a similar molecular structure to DA through its various binding sites presented by phenylalanine, offers greater sensitivity with a low detection of limit for the MIP PGE sensor for DA detection compared to the other imprinted electrode sensors that are mentioned in the literature [36,37]. Nevertheless, many related imprinted polymer electrode sensors demonstrating parallel or better limits of detection have been reported (Table 3). MIP polypyrrole (PPy)-based film was fabricated for the detection of ascorbic acid AA. The film was prepared by incorporation of a AA through the electropolymerization of pyrrole onto a PE in aqueous solution using a CV method to exhibit a high selectivity and sensitivity toward the analyte AA, and the electrode performance was detected with DPV [38]. In another study, an α-cyclodextrin (α-CD)-incorporated carbon nanotube (CNT)-coated electrode was modified and used for the simultaneous detection of EP and DA in concentration ranges of (2.0 × 10^−6^–1.0 × 10^−3^) and (1.0 × 10^−6^–1.0 × 10^−3^) mol/L, respectively, to achieve LOD around 10^−9^ M [39]. Most of the reported studies in the literature have higher limits of detection than the detection of DA by the MIP PGE sensor, even though another study was conducted on highly sensitive DA detection [35].

Furthermore, there was promising attention on using nucleic acid as an implement for driving and recognizing various compounds of interest [42], including biomolecules, such as DNA, which consists of a double helix structure, has electroactive and conductivity properties, and complexation reactions may be involved [43]. Thus, the electrochemical biosensors were commonly used recently for the analysis of DNA with other chemicals and drugs and additionally in the analysis of DNA in real samples [44]. In another study, a DNA biosensor modified with gold NPs was studied for the detection of some neurotransmitters, such as NE, by using carbon electrodes with LOD of 5 nM using the DPV method [45]. In this study, it has been confirmed that DA was successfully selected and detected among the presence of EP and NE neurotransmitters by the MIP PGE sensor with high selectivity and sensitivity (LOD: 0.193 nM). The imprinting efficiency was evaluated by calculating the imprinting factor and it was found to be 5.06. Therefore, the MIP PGE sensor used to selectively detect DA implied that the imprinting process was accomplished successfully.

## 4. Conclusions

The research area involving the methods and techniques used in DA detection is challenging and exciting, as seen from the various previous publications in this field. The electrochemical sensors are used as a favorable tool for DA detection. In this work, MIP-based electrochemical sensor modified with MAPA functional monomer was produced for the specific binding of DA molecules via the specific cavities formed on the electrode surfaces, which increased the sensitivity and selectivity for DA molecule detection, even in the presence of NE and EP molecules. In addition, AuNPs were synthesized to show an excellent development in the sensitivity and selectivity of the modified MIP electrodes. Moreover, AuNPs demonstrate their capability to accelerate the electron transfer reactions on the electrode sensor surface, enhancing the recognition of the rare oxidation peak or meaningful current signal. Currently, the modified MIPs based on PGE have confirmed they are beneficial for detecting DA in standard circumstances with 0.193 nM LOD, along with a selectivity for DA, even in the existence of interferences from NE and EP. MIP PGE has been successfully used to identify DA in human blood plasma samples. The polymer film, synthesized on the electrode surface, allows numerous benefits over normal electrode sensors for DA detection. The promising electrostatic attraction between dopamine, which is positively charged, and the modified polymer, which is negatively charged, allows for an improved analyte interaction with the electrode surface and speeds up the electron transfer mechanism. Although reduced currents were obtained during the detection of the NE and EP competitor molecules, satisfactory results were obtained for the template DA molecule. It can be believed that MIPs proved an evaluation of development acceptable enough to be applied in actual analytical applications, such as clinical and biomedical applications in the point-of-care technology devices field. The upcoming DA detection electrode sensors of the platforms or system improvements that should be costless, sensitive, and require less time to measure DA level would be very close to being everywhere within reach. This will aid in developing novel medications and methodologies, which will eventually aid in the treatment of dopamine-related disorders, such as schizophrenia, depression, Alzheimer’s disease, stress, and Parkinson’s disease.

## Figures and Tables

**Table 1:** Figures of merit of DA detection by the DPV method.

Method	^a^ R^2^	^b^ m (µA/µM)	^c^ SD (µA)	^d^ LR (nM)	^e^ LOD(nM)	^f^ LOQ (nM)
DPV	0.9955	1.0 × 10^−4^	1.102 × 10^−6^	0.395–3.93	0.193	0.581

^a^ correlation coefficient, ^b^ slope, ^c^ standard deviation, ^d^ linear range, ^e^ limit of detection, ^f^ limit of quantitation.

**Table 2:** Selectivity parameters of the MIP PGE and NIP PGE sensors for 1.32 nM DA.

MIP PGE			NIP PGE		
	Current, μA	k	Current, μA	k	k’
DA	521.25	-	103	-	-
EP	189.07	2.76	54	1.91	1.45
NE	101.24	5.15	38	2.71	1.90

**Table 3:** Different modified electrode sensors for DA detection.

Method	Material	LOD	Ref.
DPV	CO_2_ laser-induced graphene (LIG) electrodes modified with gold nanoparticles (AuNPs) and (MIP)	3.16 nM	[13]
DPV	α-cyclodextrin (α-CD)-incorporated carbon nanotube (CNT)-coated electrode	1000 nM	[34]
DPV	(MIP) electropolymer of over-oxidized polypyrrole (OPPy)	4.5 nM	[35]
CV	Molecularly Imprinted Polymer (MIP) screen-printed electrodes (SPEs)	470 nM	[36]
CV	Poly(procaterol hydrochloride) (p-ProH) membrane modified electrode.	300 nM	[40]
CV	poly (sudan III) modified carbon paste electrode (PS/MCPE)	9300 nM	[41]
DPV	MIP PGE sensor	0.193 nM	This study

## Data Availability

The data presented in this study are available on request from the corresponding author.

## Supplementary Materials

The following supporting information can be downloaded at: https://www.mdpi.com/article/10.3390/bioengineering9030087/s1, Figure S1: FTIR spectrum of non-imprinted polymer (NIP) and molecularly imprinted polymer (MIP); Figure S2: Potential of DA detection by voltammetry method in different pH of buffer solutions.
