# Supplementary material for: Gold-Modified Molecularly Imprinted N-Methacryloyl-(l)-phenylalanine-containing Electrodes for Electrochemical Detection of Dopamine
Source: Bioengineering (Basel). 2022 Feb 22;9(3):87. doi: 10.3390/bioengineering9030087 (PMC8945848; doi:10.3390/bioengineering9030087)
Supplement: Supplementary file 1 [file bioengineering-09-00087-s001.zip › bioengineering-1584527-supplementary.pdf]

Supplementary Data

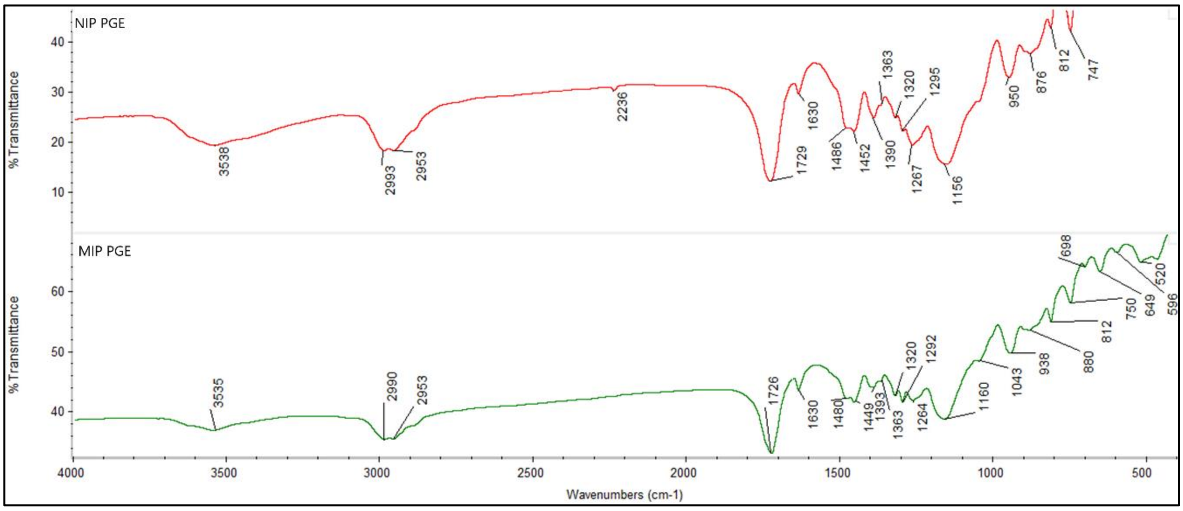

Figure S1. FTIR spectrum of non-imprinted polymer (NIP) and molecularly imprinted polymer (MIP).

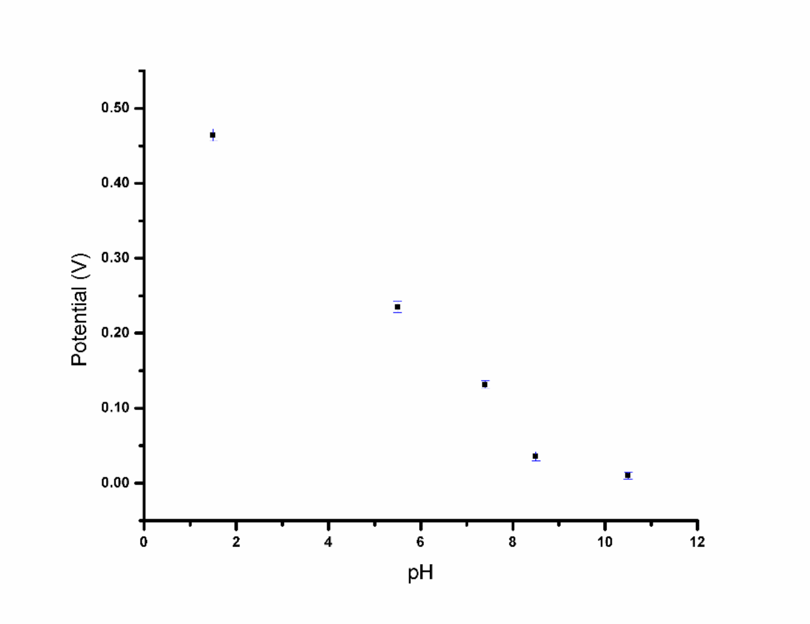

Figure S2. Potential of DA detection by voltammetry method in different pH of buffer solutions.
